# Clarification of adverse drug reactions by a pharmacovigilance team results in increased antibiotic re-prescribing at a freestanding United States children’s hospital

**DOI:** 10.1371/journal.pone.0295410

**Published:** 2024-01-12

**Authors:** Keith Feldman, Sarah L. Suppes, Jennifer L. Goldman

**Affiliations:** 1 Department of Pediatrics, Children’s Mercy Kansas City, Kansas City, MO, United States of America; 2 Department of Pediatrics, University of Missouri-Kansas City School of Medicine, Kansas City, MO, United States of America; Xiamen University - Malaysia Campus: Xiamen University - Malaysia, MALAYSIA

## Abstract

Documentation of adverse drug reactions (ADRs) is a key factor in guiding future prescribing. However, incomplete documentation is common and often fails to distinguish implicated drugs as true allergies. This in turn leads to unnecessary avoidance of implicated drug classes and may result in sub-optimal prescribing. Pharmacovigilance (PV) programs utilize a systematic approach to clarify ADR documentation and are known to improve patient safety. Yet it remains unclear if PV alters prescribing. Or, if the existence of the ADR documentation itself continues to prompt avoidance of implicated drugs. To address this, our work presents a retrospective cohort study assessing if clarification of antibiotic ADRs by a hospital-wide PV team was associated with future, safe, re-prescribing at a freestanding pediatric hospital in the midwestern United States. First, we compared the likelihood of future prescribing in an antibiotic class with an active ADR, as compared to alternative drug classes, between PV-clarified and non-clarified patients. Second, we assessed differences in adverse event rates 30-days after future prescribing based on PV clarification status. For robustness, analyses were performed on patients with ADRs in four antibiotic classes: penicillin-based beta-lactams (n = 45,642), sulfonamides/trimethoprim (n = 5,329), macrolides (n = 3,959), and glycopeptides (n = 622). Results illustrate that clarification of an ADR by PV was associated with an increased odds of future prescribing in the same drug class (Odds Ratio [95%-CI]): penicillin-based beta-lactams (1.59 [1.36–1.89]), sulfonamides/trimethoprim (2.29 [0.89–4.91]), macrolides (0.77 [0.33–1.61]), and glycopeptide (1.85 [1.12–3.20]). Notably, patients clarified by PV experienced no increase in the rate of adverse events within 30-days following the prescribing of antibiotics in the same class as an active ADR. Overall, this study provides strong evidence that PV reviews safely increase the rate of re-prescribing antibiotics even in the presence of an existing implicated drug ADR.

## Introduction

Approximately 1 in 5 patients have at least one reported adverse drug reaction (ADR) documented in their medical record [1]. Defined by the World Health Organization (WHO) as “Harmful, unintended reactions to medicines that occur at doses normally used for treatment”, the information provided by the ADR’s documentation (e.g., implicated medication, timing, reaction severity) are valuable in making future prescribing decisions and play a key role in patient safety [2]. Unfortunately, ADR documentation is often incomplete or incorrect, with inconsistencies among the history of the reaction and what details are or are not documented in the medical record [3]. Prior work has shown that more than 50% of patients with a documented allergy require a clinically relevant change in drug reaction history documentation after an interview clarifying the allergy details [4, 5].

This incomplete or inaccurate documentation of an ADR in turn results in suboptimal medication prescribing. Failure to accurately distinguish implicated drugs as hypersensitivity or side effects creates a potential for unnecessary avoidance to a preferred medication. Where use of a less desirable medication yields less effective treatment or the potential for increased side effects [6–8]. This form of ADR accuracy is particularly important in children, as incomplete or inaccurate documentation can result in years of unintended suboptimal medication prescribing.

Today, it is well known that standardized documentation protocols for ADR type and severity leads to a decrease in unnecessary drug avoidance and appropriate avoidance of contraindicated medications [9]. For instance, clarification of a penicillin allergy history, including reaction type (e.g., rash, diarrhea) and severity (e.g., mild with ability to trial similar medications, or severe requiring lifesaving interventions), has been shown in several prior works to allow for safe future prescribing of this commonly used antibiotic [10, 11]. Furthermore, the efficacy of such standardization is known to further improve through efforts of dedicated pharmacovigilance (PV) programs [12–14]. Led by trained pharmacists with a focus on ADR clarification, PV programs utilize an array of chart review, validated causality/severity measures and patient interviews to refine documentation of patient reactions and better explicate the true severity and underlying cause of potential ADRs. These programs are well established to improve patient safety. Yet it remains unclear if such a systematic approach to ADR clarification and documentation in fact alters future prescribing. Or, if despite clarification, the presence of documentation continues to prompt avoidance of implicated drugs, minimizing patient risk but continuing to result in sub-optimal prescribing [15, 16]. Given the extensive time and training required to complete PV reviews, it is critical to understand the impact of these programs on patient outcomes. This work takes a first step in providing quantitative measures of such.

Leveraging an established hospital-wide pediatric PV program, and a cohort of over 50,000 patients with documented ADRs at a freestanding midwestern pediatric hospital system, this study first assessed if ADR clarification altered future medication prescribing; comparing antibiotic re-prescribing of implicated drug classes in children with active ADRs reviewed and clarified by a PV pharmacist as compared to those with ADRs documented only by the standard of care. Further, to assess if any changes in prescribing were done safely, we present a case study assessing if re-prescribing post PV clarification was associated with increased rates of ADRs in the following 30 days.

## Materials and methods

### Study cohort

Patients for this study were identified from the Children’s Mercy Kansas City (CMKC) electronic medical record (EMR). Inclusion criteria specified patients under 18 years of age must have been admitted 1/1/2010–1/1/2020 and have an adverse reaction created in their medical record. To robustly evaluate the influence of pharmacovigilance, this study individually evaluated ADRs to four distinct classes of antibiotics. Specifically, penicillin-based beta lactams, glycopeptides, macrolides, and sulfonamides/trimethoprim. Patients without one encounter following the creation of their first ADR in a respective class were excluded, as were those who did not receive at least one future order for a systemic antibiotic while an ADR in the respective class was listed as active. All protocols were reviewed by the CMKC institutional review board (protocol #00001944). The study was deemed exempt under CFR 46.104 (d) category 4(iii) and approved with a Waiver of HIPAA Authorization. Medical record numbers and dates were used to align data but removed following analysis. Data collection and analysis took place between 10/2021 and 10/2022.

### Pharmacovigilance program

**Fig 1:**
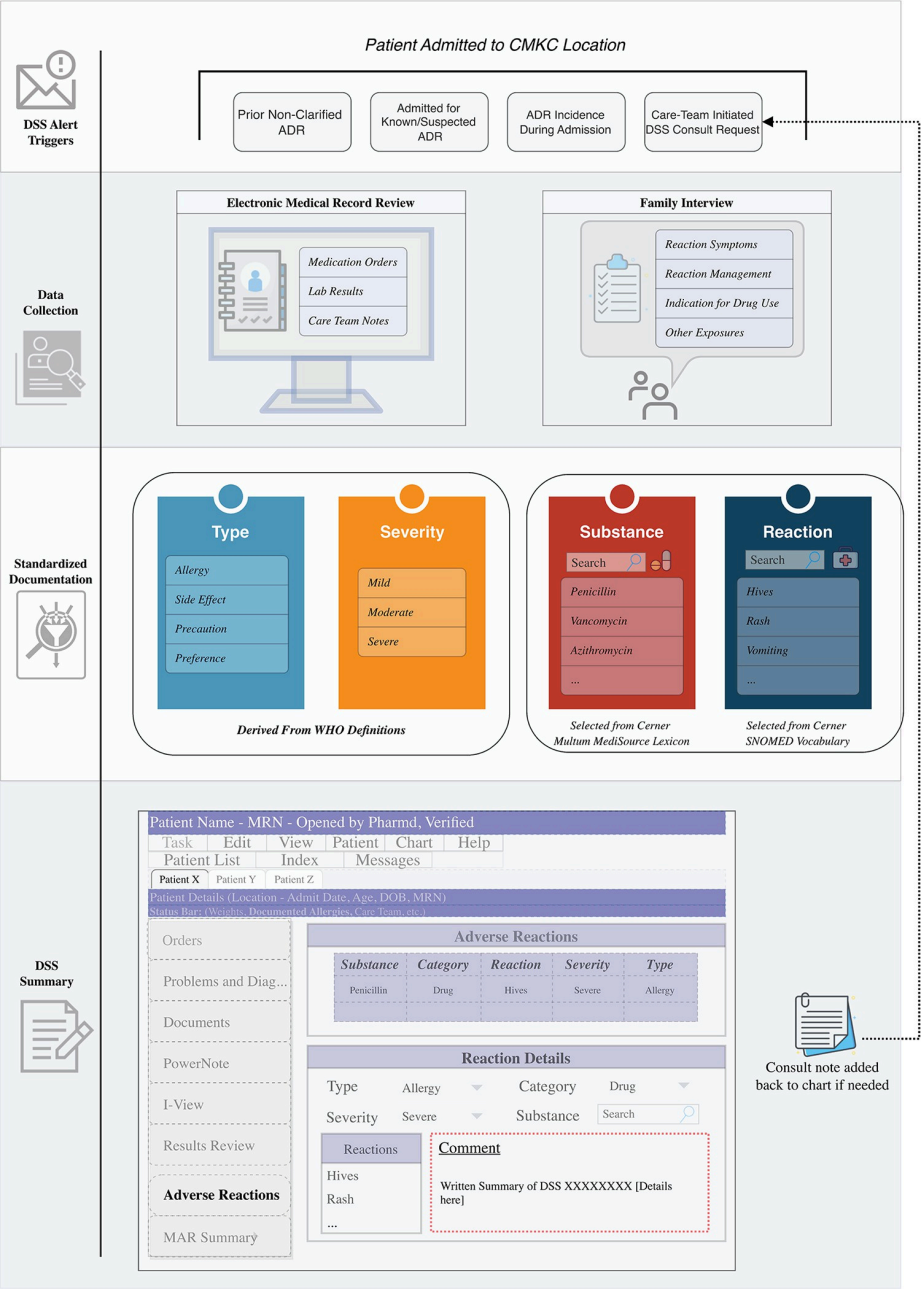
Overview of the Children’s Mercy Kansas City pharmacovigilance team’s review and clarification process.

CMKC has maintained an active PV program since October 2010. The team, led by a specialized pharmacist, provides review of admitted patients with documented ADRs or through consults initiated by the care team. Utilizing both interviews and EMR review, the PV team performs extensive data collection for each ADR. These data are then used to complete standardized documentation efforts including the Naranjo causality score (*and associated classification*: *≥9 Definite*, *5–8 Probable*, *1–4 Possible*, *≤0 Doubtful*) for the implicated drug, and clarification of the type and severity of the reaction. This information is recorded in a patient’s EMR, and a written summary of the reaction is attached to the ADR record. An overview of this process can be found in Fig 1.

### Data

For included patients, data were extracted across three domains: ADR documentation, medication prescribing, and encounter details.

#### ADR documentation

All details surrounding patients’ ADRs were extracted. Including date/time the documentation was recorded in the EMR, its severity, reaction type, and medication class. Over the lifespan of the EMR entry, updates to any field were extracted independently with a unique identifier and update time to allow for temporal analysis. Reactions found to be assigned with multiple medical records (n = 99, < .01%) were excluded. Adverse reactions to substances other than “drug” (e.g., food) were removed. All remaining ADRs were included for analysis, as exclusion based on a specific causality certainty (e.g., *Definite*), would require universal use of the validated Naranjo tool, only available for PV clarified patients. Filtering the PV cohort without similar filtering to the general non-clarified cohort could introduce bias, as a subset the unclarified ADR is expected to also have categories of higher uncertainty (e.g., probable, possible) had they been labeled.

*Pharmacovigilance review*. Any written comments pertaining to an ADR (entered by physician, pharmacovigilance team, care team, etc.) were extracted as available. PV team comments were then isolated, reviews by the team are marked with the specific terms (“Drug Safety Service”, and analogous “DSS”, as well as “IPT” representing the Individualized Pediatric Therapeutics program).

*Drug class*. The active substance for each ADR, were mapped to the Anatomical Therapeutic Chemical (ATC) Classification System–a classification system maintained by the WHO [17]. To link the implicated drug for a given ADR to ATC class, we utilized the RxNorm ontology maintained by the National Library of Medicine [18] specifically queried from the RxNav API using the pharmpy package. Mapping was performed using the API data as queried on October 2^nd^, 2023.

Adverse reactions could also be associated with a more general substance (e.g., “Augmentin Tablets”). In these cases, the substance was linked to an ingredient through Cerner Millennium reference tables. National Drug Code (NDC) codes for all drugs containing that ingredient were obtained and mapped to an ATC class. For a small subset (~22) of substances that could not be matched, manual review was performed by a pharmacist (S1 Table). When identifying an ADR in any of the four given antibiotic classes ATC codes were always treated as an ontology, for which more specific classes (e.g., ATC class JO1CA—*penicillins with extended spectrum*) were considered valid at a higher level (e.g., JO1C).

#### Medication prescribing

All orders for each included patient were extracted from the CMKC EMR during the study period. For each order, the drug name, route, and order type (e.g., modification, initial entry) were extracted along with the date/time the order was created. All non-systemic routes, orders that specified a “flush”, and modification orders were excluded. Similar to the implicated ADR drug, all orders were mapped to their respective ATC class. Orders that were unable to be matched through the RxNorm system (either through NDC or RxCUI) were removed, however a review of the top 50 most frequent unmatched items yielded no antibiotics.

#### Encounter details

For each patient, the date/time for every encounter at CMKC hospital and outpatient clinics were extracted during the study period. As well as a complete list of all diagnoses from each encounter.

## Data analyses

To examine the association between PV review and antibiotic prescribing in patients with an active ADR, this manuscript presents two distinct analyses. First, quantifying differences in likelihood between prescribing a drug within the same class as an active ADR versus an alternative antibiotic class. Second, presenting a case study of ADR rates following prescribing in the same drug classes based on the clarification status. Each analysis was repeated independently with each of the four listed ATC classes.

### Patterns of re-prescribing

For a given cohort, we first derived several metrics for each of a patient’s drug orders following the date of when their first ADR was documented. These included both: a binary indicator if the ordered drug falls within the respective ATC class (representing a patient’s ADR), or an alternative antibiotic class, as well as a binary indicator if PV had reviewed the ADR. As patients can have multiple ADRs documented for the same drug class, clarification was defined as prior (occurring before the order date) review of any active ADR in the respective drug class.

We then aggregated data at the patient level, representing those patients for which no orders were clarified and those with at least one order following PV clarification of an active ADR. For each group we identified if a drug in the same drug class was re-prescribed after an active ADR was first documented. For individuals without PV clarification this assessed all orders, where for those with PV assessment, only orders following clarification were considered.

To assess the differences in odds of having a drug prescribed in an active ADR class between patients whose reactions were clarified and those who were not, we utilized a 500-iteration bootstrapped logistic regression. In this approach, data is resampled from the study cohort, with replacement, and the model is refit. At each iteration, model coefficients are stored, and results are presented as a mean coefficient value, and 95% confidence internal as determined by the 2.5% and 97.5 percentiles from the sampling distribution. This approach is known to provide a significantly more robust estimate of expected direction and magnitude of associations for a population, as compared to a singular model fit.

In all iterations, the model was adjusted for known confounders for re-prescribing including age in years at time ADR was created, patient sex, race. Further, to help account for potential bias of patients with more opportunity for re-prescribing, the model was also adjusted for the total number of future encounters each patient has in the CMKC system following the documentation their an ADR for the respective class. A binary indicator for PV clarification status was used, reference level–not clarified.

### Case study: Prescribing safety

Our second analysis was designed to assess the safety of prescribing antibiotics in the same class as an active antibiotic allergy, defined as the occurrence of ADRs within 30 days of a given order and compared between those patients clarified by PV and those not clarified.

To do so, we derived a binary factor to indicate if clarification occurred prior to each order. We then extracted all clinical diagnoses from encounters occurring within the following 30 days, inclusive of the encounter in which the drug was ordered. Diagnoses were then labeled with a binary indicator representing an ADR as described by Holh et al. [19] Specifically, we focus on the most direct classifications A1 (Induced by medication) and severity B1 (Poisoning by medication). Given the focus on antibiotic ADRs, this list was refined by a PV trained pharmacist to relevant events (complete list used for this manuscript can be found in S2 Table). ADRs identified by these codes were then subjected to detailed chart review to assure the reaction was a true ADR and associated with the implicated antibiotic order, and not a concurrent medication.

As in the re-prescribing analysis, data were then aggregated to the patient level. For non-clarified patients, we assessed if any future order in the respective class was associated with an ADR, and for those clarified by PV we focus on the order made after clarification. Given the extremely low prevalence of ADRs, a Fisher’s Exact test was used.

Of note, to differentiate ADRs from other non-specific forms of illness, the ADR diagnoses defined by Holh et al. are defined only for ICD-10 nomenclature. As such we focused on a subset of patients whose first ADR in the respective class occurred following our hospital’s shift to ICD-10 in October 2015. To allow for a buffer during transition, a threshold of 11/1/2015 was used. Further, in an effort to provide the most robust assessment, we required patients in this case have their first allergy in the respective class created after the 11/2015 date. This was done in an attempt to minimize bias from potential earlier exposures to the drug that could not be flagged by the ADR codes, which may in turn influence odds of receiving the drug again knowing ADRs would be unlikely.

## Results

**Fig 2:**
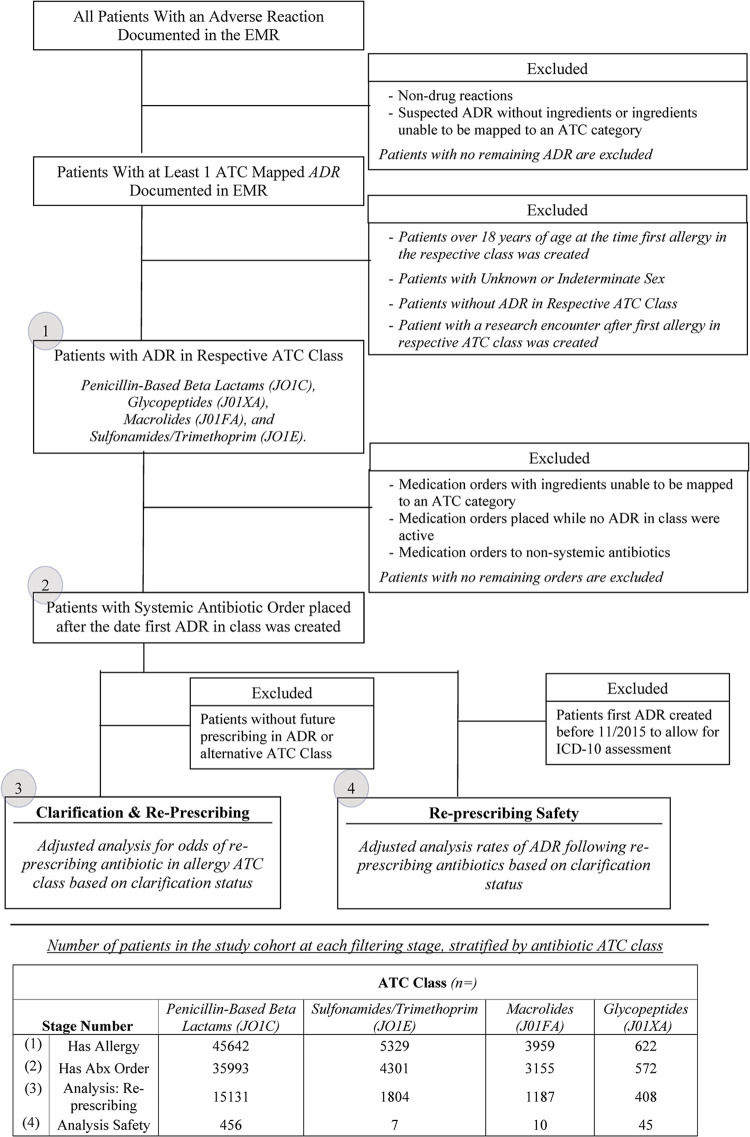
Patient selection flow diagram. Figure (above) presents the inclusion and exclusion criteria for the study cohort for each of the two primary analyses. The table (below) presents the specific count of patients at each stage across the four ATC classes analyzed.

In total, 83,295 patients had a documented ADR (with an associated NDC) over the 10-year study period, representing 114,049 unique ADR entries. Specific ADRs evaluated included penicillin-based beta-lactams (n = 45,642), sulfonamides/trimethoprim (n = 5,329), macrolides (n = 3,959), and glycopeptides (n = 622). A review of the exclusion criteria for the cohort and for each analysis can be found in Fig 2 along with the respective counts at each stage. An overview of demographics for the final study cohorts can be found in Table 1.

**Table 1:** Study cohort demographics for each of the four evaluated ATC classes.

		Penicillin-Based Beta Lactams (JO1C)		Sulfonamides/Trimethoprim (JO1E)		Macrolides (J01FA)		Glycopeptides (J01XA)	
		**Clarified**	**Not Clarified**	**Clarified**	**Not Clarified**	**Clarified**	**Not Clarified**	**Clarified**	**Not Clarified**
n =		1437	13694	282	1522	151	1036	212	196
Years of Age, mean (SD)		7.4 (5.5)	6.1 (4.9)	9.7 (5.5)	9.0 (5.2)	9.6 (5.2)	8.0 (5.1)	9.4 (5.5)	9.2 (5.5)
Sex, n (%)	*Female*	667 (46.4)	6782 (49.5)	157 (55.7)	955 (62.8)	85 (56.3)	536 (51.7)	91 (42.9)	99 (50.5)
	*Male*	770 (53.6)	6912 (50.5)	125 (44.3)	567 (37.3)	66 (43.7)	500 (48.3)	121 (57.1)	97 (49.5)
Race, n (%)	*Hispanic*	170 (11.8)	1702 (12.4)	23 (8.2)	128 (8.4)	9 (6.0)	83 (8.0)	24 (11.3)	25 (12.8)
	*Black*	113 (7.9)	1316 (9.6)	23 (8.2)	134 (8.8)	10 (6.6)	61 (5.9)	7 (3.3)	17 (8.7)
	*White*	1068 (74.3)	9839 (71.9)	213 (75.5)	1182 (77.7)	126 (83.4)	836 (80.7)	168 (79.3)	135 (68.9)
	*Other*	86 (6.0)	837 (6.1)	23 (8.2)	78 (5.1)	6 (4.0)	56 (5.4)	13 (6.1)	19 (9.7)
# of Future Encounters, mean (SD)		31.7 (41.6)	13.9 (19.5)	47.8 (60.8)	17.6 (24.2)	40.2 (49.5)	16.2 (23.9)	64.7 (65.9)	32.5 (50.4)

### Patterns of re-prescribing

**Fig 3:**
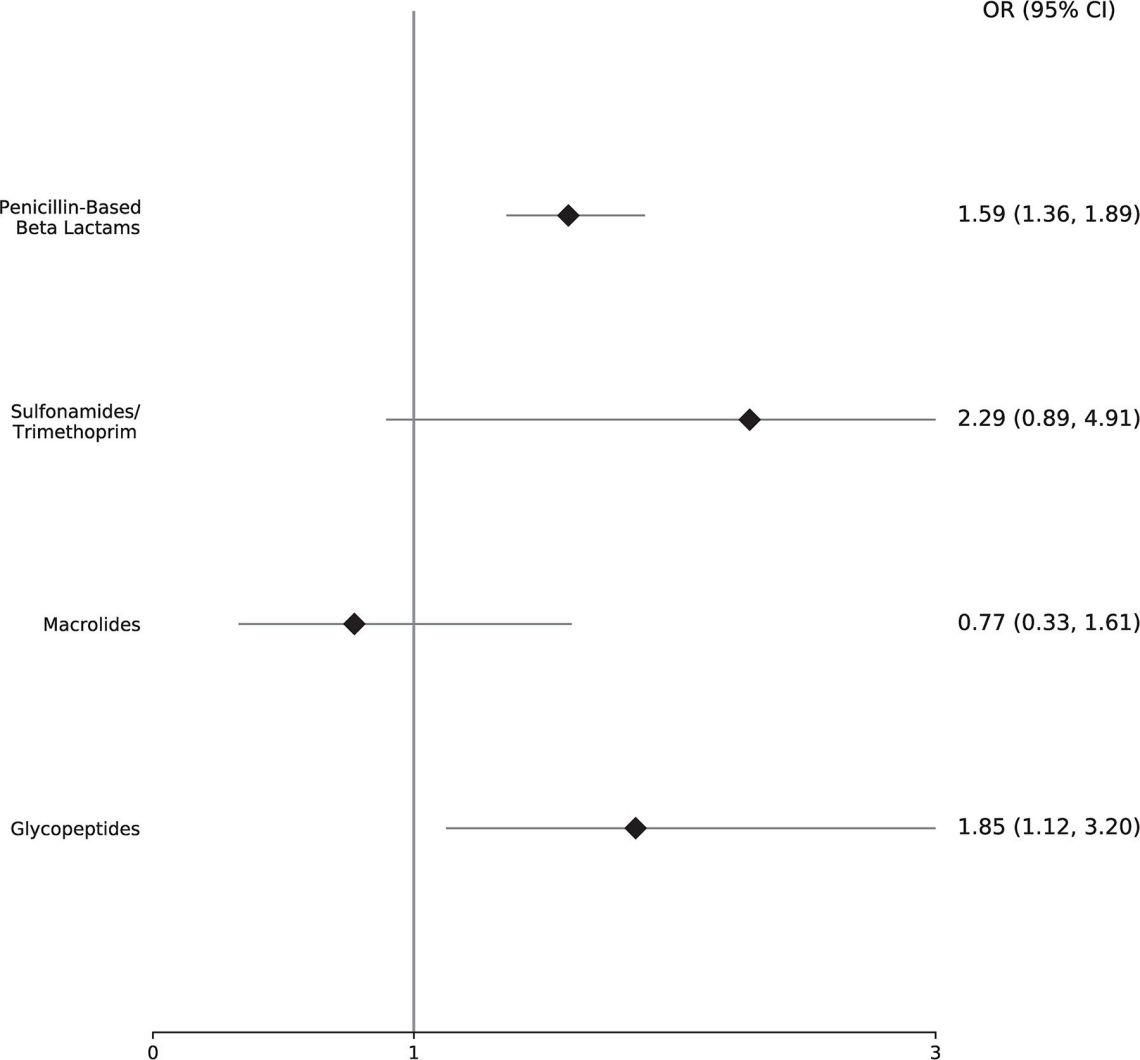
Mean Odds ratio (OR) and 95% confidence intervals from the bootstrapped regression. OR >1 represent an increased odds of receiving a future drug order in the same ATC class as an active allergy for patients whose allergy was clarified by Children’s Mercy Kansas City pharmacovigilance as compared to those who were not. Complete sampling distributions for the OR across all bootstrap iterations can be found in S1 Fig.

Fig 3 presents the odds ratio (and associated 95% conference interval) between clarified versus non-clarified patients for future re-prescribing in each of the four evaluated drug classes. Beta-lactam and glycopeptide were associated with increased odds of prescribing a drug in the same class, as compared to an antibiotic in a different ATC class. Looking next to sulfonamides/trimethoprim and macrolide ADRs, we note a positive and negative association with re-prescribing, respectively. However, both present with extremely wide confidence intervals, suggesting a high degree of heterogeneity in the impact of PV on patients with reactions in these drug classes. To determine if PV has any meaningful or consistent impact on all or a subgroup of these patient’s additional work is needed, particularly that describing specific patient indication at the time of future medication orders.

Finally, we recognize, that avoidance of the drug class may be warranted after clarification, and thus may result in a lower proportion of prescribing in that same drug class. To strengthen the conclusions drawn from this analysis, we turn to our secondary analysis, looking again to penicillin-based beta-lactam (ATC: JO1C) ADRs, but restricting the alternative class to “other beta-lactam antibacterial”: (ATC: JO1D, e.g., cephalosporins). In this way, we specifically analyzed those individuals who received a future beta lactam, while a *penicillin-based* ADR was active to assess how PV review may have modified prescribing to known alternatives (i.e., cephalosporins). In doing so, we again note increased odds of prescribing within the clarified group, Odds Ratio (Confidence Interval) 1.79 (1.50–2.16).

### Case study: Prescribing safety

Across the four antibiotic classes, 518 patients received an order for a drug in the same class of an active ADR. In total the ICD code set identified 23 patients with an ADR event. Chart review of these found only 3 to represent true reactions following an order, while the others were associated with concurrently administered drugs or drug challenge events that although successfully passed were coded as adverse effects given their induction of a potential reaction. A breakdown of patients by drug class and clarification status can be found in Table 2, as well as the mean number of unique days per patient on which a drug in the class was ordered, to provide a sense of magnitude for how many prescribing events were evaluated for potential ADRs. Note, this value was aggregated at the day-level, as the ADR rate was evaluated in a 30-day window following an order and thus multiple orders on the same day produce the same ADR results.

**Table 2:** Summary of ADR events in the 30-days following prescribing a drug in the same class as an active ADR by drug-class. *p-values calculated from Fisher’s Exact Test are approximately equal to 1.0 in software but are more accurately presented as 0.9999.

Drug class	Number patients	Total orders in implicated class	Mean number of orders in implicated class per patient	ADR breakdown by clarification status		
Penicillin-based beta-lactams	456	681	1.2		No ADR	ADR
				Clarified	45	0
				Non-Clarified	410	1
				*p~ = 0.999		
Sulfonamide/Trimethoprim	7	57	2.3		No ADR	ADR
				Clarified	3	0
				Non-Clarified	4	0
				*p~ = 0.999		
Macrolide	10	21	1.3		No ADR	ADR
				Clarified	1	0
				Non-Clarified	9	0
				*p~ = 0.999		
Glycopeptide	45	232	3.73		No ADR	ADR
				Clarified	25	2
				Non-Clarified	18	0
				p = 0.51		

In all classes, we note no significant difference between rates of ADRs for clarified and non-clarified patients. As we excluded patients for which any prior ADR in the drug class had been created prior to 11/2015, we are confident this represents the first re-prescribing event in the CMKC system.

A review of the 3 events, we find the single penicillin-based beta-lactam reaction occurred in a patient with a documented ADR involving penicillin. The patient was never clarified by PV and approximately 1 year following the creation of the original ADR report patient received amoxicillin in the CMKC emergency department. Approximately 7-days after this encounter the child presented to CMKC urgent care for a rash determined to be a reaction to the amoxicillin. The two glycopeptide reactions represented infusion-related reactions. In both cases, patients with a documented allergy to vancomycin were re-prescribed the drug. Both patients were clarified by PV, in their documentation noting the reaction was not life threatening and could be mediated with pre-treatment of Benadryl. These two patients’ experienced vancomycin flushing syndrome, neither infusion was stopped and both patients received future courses.

## Discussion

ADR documentation in the medical record ideally provides care teams with information to understand potential risks for adverse events (expected reaction, severity, etc.) associated with prescribing a drug, or class of drugs. Incomplete, or insufficiently detailed, documentation has presented a concern for patient safety, and been a primary driver for avoidance of future prescribing in implicated drug classes resulting in sub-optimal prescribing [20–22]. By standardizing approaches to documentation, PV teams have achieved significant improvements in patient safety [23, 24]. However, such results entangle multiple concepts, where safety can be achieved by either continued avoidance of implicated drugs or through a combination of safe re-prescribing and appropriate avoidance [25]. As PV programs continue to grow, more information is needed to understand the impact PV has on future prescribing practices.

Data, primarily with the penicillin drug class, demonstrated that by clarifying the ADR history, re-prescribing the same medication can often be done safely in patients with a prior history of a low-risk adverse reaction [10, 26]. Taking this further, our study evaluated the impact of our hospital-wide PV program in a pediatric hospital and the association of ADR clarification and documentation compared to standard of care documentation. Our findings provide evidence that ADR review and documentation by the PV program was associated with different re-prescribing patterns as compared to those without PV documentation with more re-prescribing of same drug class in patients with PV documentation.

It is notable, however, that effect sizes between the four evaluated drug classes varied significantly. In line with prior studies, PV clarification was found to have a strong and consistent association with increased re-prescribing for penicillin-based beta-lactams ADRs. Encouragingly, we see similar patterns with sulfonamides/trimethoprim and glycopeptides, however with wider confidence bounds for the magnitude of the effect, suggesting PV clarification may be heterogenous and vary by latent unaccounted demographic or clinical factors. With macrolides, no difference in prescribing was observed, again suggesting that the interface between ADR clarification and prescribing is complex.

Critically, this work illustrates that despite increased rates of re-prescribing of the same or similar drug classes, there was not an association to increased likelihood of an adverse event following prescribing for those clarified by PV. Together these results suggest, increased rates of re-prescribing occur safely, and appropriate avoidance of severe ADRs likely occurred. Based on the overall high prevalence of patient ADR labels, PV programs may be an effective approach to optimize prescribing.

It is worth noting the extensive review conducted by PV teams is labor intensive and time consuming, requiring effort from both the PV team and the patient/family. Current work by our team is underway to identify specific elements within PV ADR clarification and/or documentation that differentiate those patients who are safely re-prescribed a drug to optimize future PV efforts. This work is directly aligned with contemporary efforts to utilize computational methods for precision pharmacovigilance [27]. Concurrently, we recognize, that given a finite labor force and time, there remains a need to quantify those patient profiles most likely to benefit from PV review. This manuscript takes the first step in doing so, we hope it may act as an evaluation framework for a broader set of ADR classes. Allowing for more insight into potential variability by reaction type, timing, and clinical profile of a patient.

### Limitations

When interpreting the results of this study it is important to note several limitations. First, with respect to the cohort itself, the analysis represents practice at a single center and thus may not generalize to institutions more broadly. Further, although comparison of future re-prescribing requires all patients to return for a separate encounter of any type and receive an antibiotic order, the clarified cohort may itself be biased with more severe medical conditions and thus impact overall prescribing. Second, with respect to the retrospective study design, data utilized for analysis covers over 10,000 unique orders across the four evaluated ATC classes and as such it was not feasible to assess indication for each future order. In turn presenting potential for bias in cases where re-prescribing may not be optimal even after clarification. It is also possible concurrent medications could create contraindications, thus influencing prescribing. However, consistent results within our sub-analysis assessing re-prescribing of penicillin-based beta-lactams versus other beta lactams suggest these effects to be minimal. Finally, use of ICD codes to identify adverse events is known to lack sensitivity. The decision to utilize published standards was done to remove bias from our own institutional practice, however we recognize assessment of future adverse events may underestimate the total incidence.

## Conclusion

This study demonstrated that a hospital-wide PV program was associated with changes in medication prescribing practices, resulting in those with an ADR label to be re-prescribed the same drug class without increased rates of adverse drug events. Given the high rates of poor ADR documentation, additional work is needed to understand how PV programs can most effectively and efficiently support ADR clarification and documentation to promote future safe medication prescribing.

## Supporting information

S1 Table Mapping of unmatched reaction substance descriptions to NDC codes. (DOCX)

S2 Table Relevant ICD-10 codes for antibiotic ADRs–Adapted from Holh et al. (2014). (XLSX)

S1 Fig Bootstrap distribution of re-prescribing analysis by drug class. (PDF)

S1 Data De-identified CSV files for prescribing analysis. (ZIP)
