# Supplementary material for: Clarification of adverse drug reactions by a pharmacovigilance team results in increased antibiotic re-prescribing at a freestanding United States children’s hospital
Source: PLoS One. 2024 Jan 12;19(1):e0295410. doi: 10.1371/journal.pone.0295410 (PMC10786368; doi:10.1371/journal.pone.0295410)
Supplement: S1 Table — (DOCX) [file pone.0295410.s001.docx]

| **Description** | **NDC** | **Mapped Drug** |
| --- | --- | --- |
| sulfa drug | 49708-145-01 | sulfamethoxazole 400 MG / trimethoprim 80 MG Oral Tablet [Bactrim] [RxCUI = 208416] |
| Sulfa drug | 49708-145-01 | sulfamethoxazole 400 MG / trimethoprim 80 MG Oral Tablet [Bactrim] [RxCUI = 208416] |
| sulfa drugs | 49708-145-01 | sulfamethoxazole 400 MG / trimethoprim 80 MG Oral Tablet [Bactrim] [RxCUI = 208416] |
| Sulfonamide antibiotic adverse reaction | 49708-145-01 | sulfamethoxazole 400 MG / trimethoprim 80 MG Oral Tablet [Bactrim] [RxCUI = 208416] |
| Sulfonamides Causing Adverse Effects in Therapeutic Use | 49708-145-01 | sulfamethoxazole 400 MG / trimethoprim 80 MG Oral Tablet [Bactrim] [RxCUI = 208416] |
| sulfadoxine | 49708-145-01 | sulfamethoxazole 400 MG / trimethoprim 80 MG Oral Tablet [Bactrim] [RxCUI = 208416] |
| metronidazole | 00025-183-131 | metronidazole 250 MG Oral Tablet [Flagyl] [RxCUI = 207287] |
| penicillin | 54771-313-901 | 10 ML novobiocin 40 MG/ML / penicillin G procaine 20000 UNT/ML Prefilled Syringe [Albadry] [RxCUI = 1484873] |
| Penicillin | 54771-313-901 | 10 ML novobiocin 40 MG/ML / penicillin G procaine 20000 UNT/ML Prefilled Syringe [Albadry] [RxCUI = 1484873] |
| Augmentin Tablets | 55289-024-209 | amoxicillin 250 MG / clavulanate 125 MG Oral Tablet [Augmentin] [RxCUI = 824186] |
| amoxicillin | 51311-020-350 | amoxicillin 100 MG Oral Tablet [Biomox] [RxCUI = 791942] |
| Amoxicillin | 51311-020-350 | amoxicillin 100 MG Oral Tablet [Biomox] [RxCUI = 791942] |
| Amoxicillin + clavulanate | 86136-000-221 | amoxicillin 100 MG / clavulanate 25 MG Oral Tablet [Betacillin] [RxCUI = 2566438] |
| Amoxicillin/Clavulanate | 86136-000-221 | amoxicillin 100 MG / clavulanate 25 MG Oral Tablet [Betacillin] [RxCUI = 2566438] |
| clavulanate | 86136-000-221 | amoxicillin 100 MG / clavulanate 25 MG Oral Tablet [Betacillin] [RxCUI = 2566438] |
| cefdinir | 00093-316-006 | cefdinir 300 MG Oral Capsule [RxCUI = 200346] |
| tazobactam | 00206-885-208 | Zosyn (piperacillin / tazobactam) 2.25 GM Injection [RxCUI = 1659134] |
| Cephalexin | 52959-008-720 | Keflex 500 MG Oral Capsule [RxCUI = 212339] |

Supporting Table S1: Mapping of Unmatched Reaction Substance Descriptions to NDC Codes
