# Supplementary material for: Clarification of adverse drug reactions by a pharmacovigilance team results in increased antibiotic re-prescribing at a freestanding United States children’s hospital
Source: PLoS One. 2024 Jan 12;19(1):e0295410. doi: 10.1371/journal.pone.0295410 (PMC10786368; doi:10.1371/journal.pone.0295410)
Supplement: S1 Fig — (PDF) [file pone.0295410.s003.pdf]

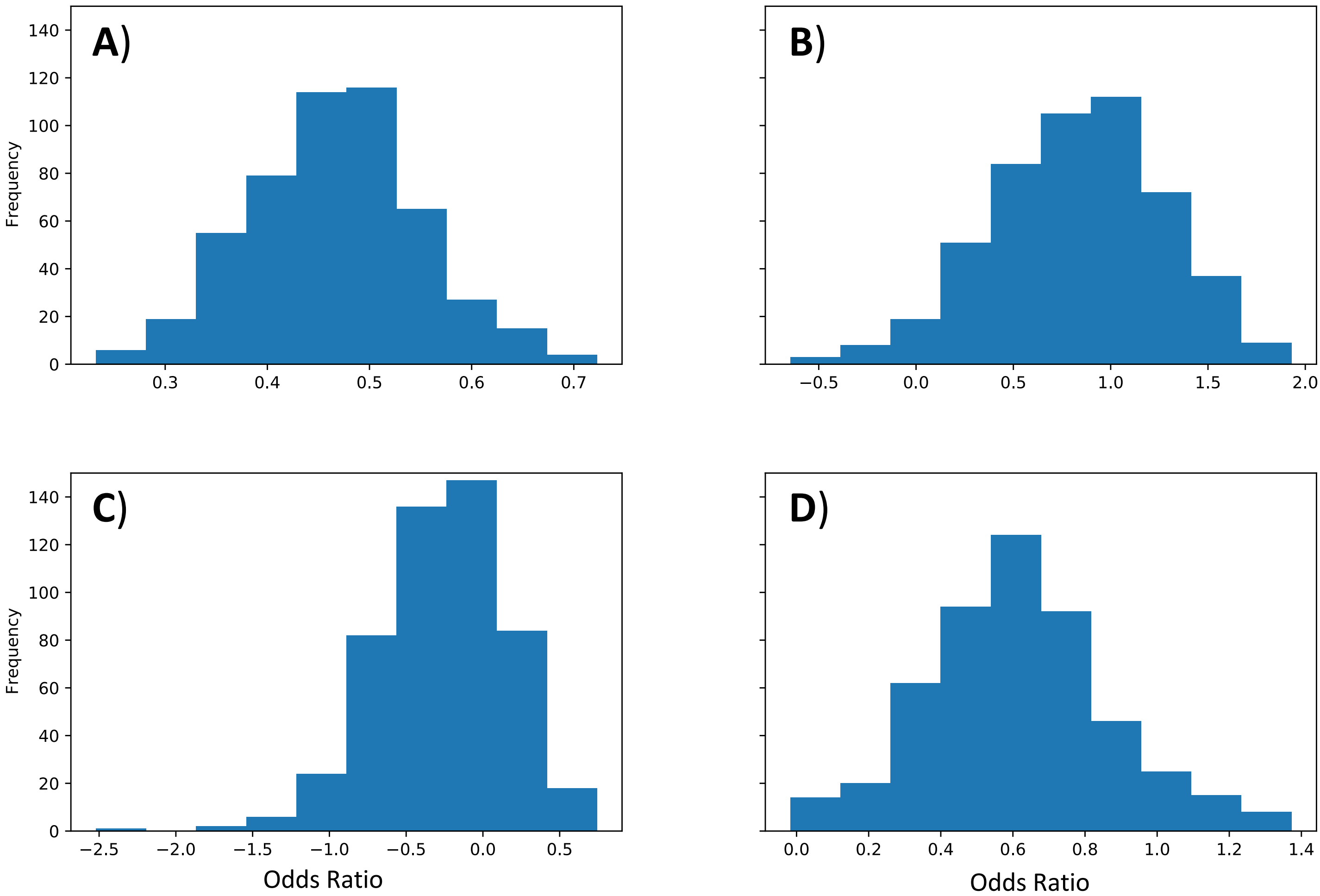

Supporting Fig S1: Sampling distribution for the OR coefficient across the bootstrap iterations of each drug class. OR >1 represent an increased odds of receiving a future drug order in the same ATC class as an active allergy for patients whose allergy was clarified by Children's Mercy Kansas City pharmacovigilance as compared to those who were not.

A) Penicillin-Based Beta Lactams (JO1C), B) Sulfonamides/Trimethoprim (JO1E), C) Macrolides (J01FA), and D) Glycopeptides (J01XA)
